# Biological pretreatment with white rot fungi for preparing hierarchical porous carbon from Banlangen residues with high performance for supercapacitors and dye adsorption

**DOI:** 10.3389/fmicb.2024.1374974

**Published:** 2024-05-30

**Authors:** Wen Kong, Xinyu Zhang, Xiao Fu, Can Zhou, Linjiang Fan, Wanju Zhang

**Affiliations:** Hubei Key Lab for Processing and Application of Catalytic Materials, LiShizhen College of Traditional Chinese Medicine, Huanggang Normal University, Huanggang, China

**Keywords:** white rot fungi, pretreatment, traditional Chinese medicine residue, porous carbon, supercapacitors, adsorbent

## Abstract

White rot fungi possess superior infiltrability and biodegradability on lignocellulosic substrates, allowing them to form tailored microstructures which are conducive to efficient carbonization and chemical activation. The present research employed white rot fungus pretreatment as a viable approach for preparing porous carbon from Banlangen residues. The resultant F-A-BLGR-PC prepared by pretreating Banlangen residues with white rot fungi followed by carbonization and activation has a hierarchical porous structure with a high specific surface area of 898 m^2^ g^−1^, which is 43.4% greater than that of the unprocessed sample (R-BLGR-PC). When used as an electrode for supercapacitors, the F-A-BLGR-PC demonstrated a high specific capacitance of 308 F g^−1^ at 0.5 A g-1 in 6 M KOH electrolyte in three-electrode configuration. Moreover, the F-A-BLGR-PC based symmetric supercapacitor device achieved a superb cyclic stability with no obvious capacitance decay after 20,000 cycles at 5 A g^−1^ in 1 M Na_2_SO_4_ electrolyte. Additionally, the F-A-BLGR-PC sample was found to be an ideal adsorbent for removing methyl orange (MO) from water, exhibiting an adsorption ability of 173.4 mg g^−1^ and a maximum removal rate of 86.6%. This study offers a promising method for the preparation of a porous carbon with a high specific surface area in a biological way using white rot fungi pretreatment, and the derived carbon can not only be applied in energy storage but also in environmental remediation, catalysis, and so on.

## 1 Introduction

Traditional Chinese Medicine Residue (TCMR) is a solid substance generated by the extraction of pharmaceutical ingredients from medicinal plant materials. Generally, TCMR is lost as garbage, which not only leads to the waste of resources, but also causes potential environmental pollution (Ma et al., 2019; Zhou et al., 2019). Isatidis Radix (also known as Banlangen, BLG in Chinese) is one of the most commonly used traditional edible-medicinal herbs for detoxification for thousands of years. It has been traditionally applied in traditional Chinese medicine to treat influenza and other viral infectious diseases in clinics for centuries. A large amount of pharmaceutical waste is produced in the production process of BLG drugs (Xu et al., 2016). In this regard, developing processing technique transforming pharmaceutical waste to high value-added products is highly required.

Alternatively, the residue of BLG can be used as biomass source for the fabrication of porous carbon (AC) since biomass derived AC has been a hot topic and various biomass including bio-waste were utilized as starting material of the synthesis of AC (Shao et al., 2020, 2022b). Traditionally, biomass derived AC was conducted by direct carbonization and activation of carbonaceous sources with various activators like KOH, ZnCl_2_, H_3_PO_4_, etc. (Tian et al., 2020). In fact, the pristine microstructure of biomass sources was proved to be a key factor influencing the pore structure, surface chemistry and the performances of the resultant AC. As such, some chemical pretreatment methods were reported to tune the inherent structure of the bio-sources (Liu et al., 2019; Wan et al., 2020). However, pretreating the adopted biomass by a biological method is rarely reported. Because of the cell wall of secondary plants is mainly composed of cellulose, hemicellulose, and lignin, where cellulose/hemicellulose is usually deposited by hemicellulose/lignin, resulting in a rigid structure and discontinuous channels between cellulose or hemicellulose and the external environment (Zhang et al., 2019; Rawat et al., 2022; Shao et al., 2022a). This can limit the efficiency of carbonization and chemical activation processes. Drawing inspiration from the way microorganisms break down lignocellulose in nature, it is reasonable that fungi may be able to generate layered porous structures. Among all the fungi, white rot fungi are known for their powerful ability to break down lignocellulose, which can be used to create loosely structured lignocellulosic precursors (Martínez et al., 2005; Bugg et al., 2011; Floudas et al., 2012). These precursors are ideal for efficient carbonization and chemical activation.

Herein, we used BLG residue (BLGR) as a raw material and *P. vitreus* to ferment and pretreatment fungi to get the as-made starting material, in which the mycelium of these fungi spreads quickly in three-dimensional space along the cell wall of plants (Chen et al., 2022; Lin et al., 2022), and has a strong mechanical interpenetrating capacity. After being degraded by white rot fungi, TCMR become softer and more pliable, creating a favorable microbial “pre pore” for the preparation of ultra-high specific surface area porous carbon materials. The as-made BLGR was further carbonized and activated at high temperature to obtain the final porous carbon. The final obtained product owns high surface area, rich oxygenic functionalities and exhibit superior supercapacitor and sorption performances. The present study not only show a simple and eco-friendly technique, but also suggest that fungi pretreatment may be a viable option for creating high-value carbon materials from TCMR.

## 2 Materials and methods

### 2.1 Fungus and inoculum preparation

White rot fungus *Physisporinus vitreus* was kindly provided by the Institute of Microbiotechnology of Environment and Resources, Huazhong University of Science and Technology. *P. vitreus* was cultured on potato extract agar slants for 7 days at 28°C. Five disks (1 cm in diameter) of inocula were grown in potato extract broth for 5 days (150 rpm, 28°C).

### 2.2 Fungal pretreatment

BLGR was collected from Qichun County, Hubei Province, China, and dried at 60°C in an oven for 2 days. The raw BLGR was ground to pass through an 80-mesh sieve. Biological pretreatments with *P. vitreus* were carried out in 250-mL Erlenmeyer flasks containing 10 g of BLGR and 15 mL of distilled water. The flasks were sterilized in an autoclave for 30 min at 121°C and aseptically inoculated with 5-mL fungal inocula. The cultures were maintained statically at 28°C for one to three weeks, and then dried at 60°C for 2 days for the preparation of porous carbon. A set of non-pretreated, sterilized BLGR was used as a control.

### 2.3 Synthesis of porous carbon derived from BLGR

**SCHEME 1:**
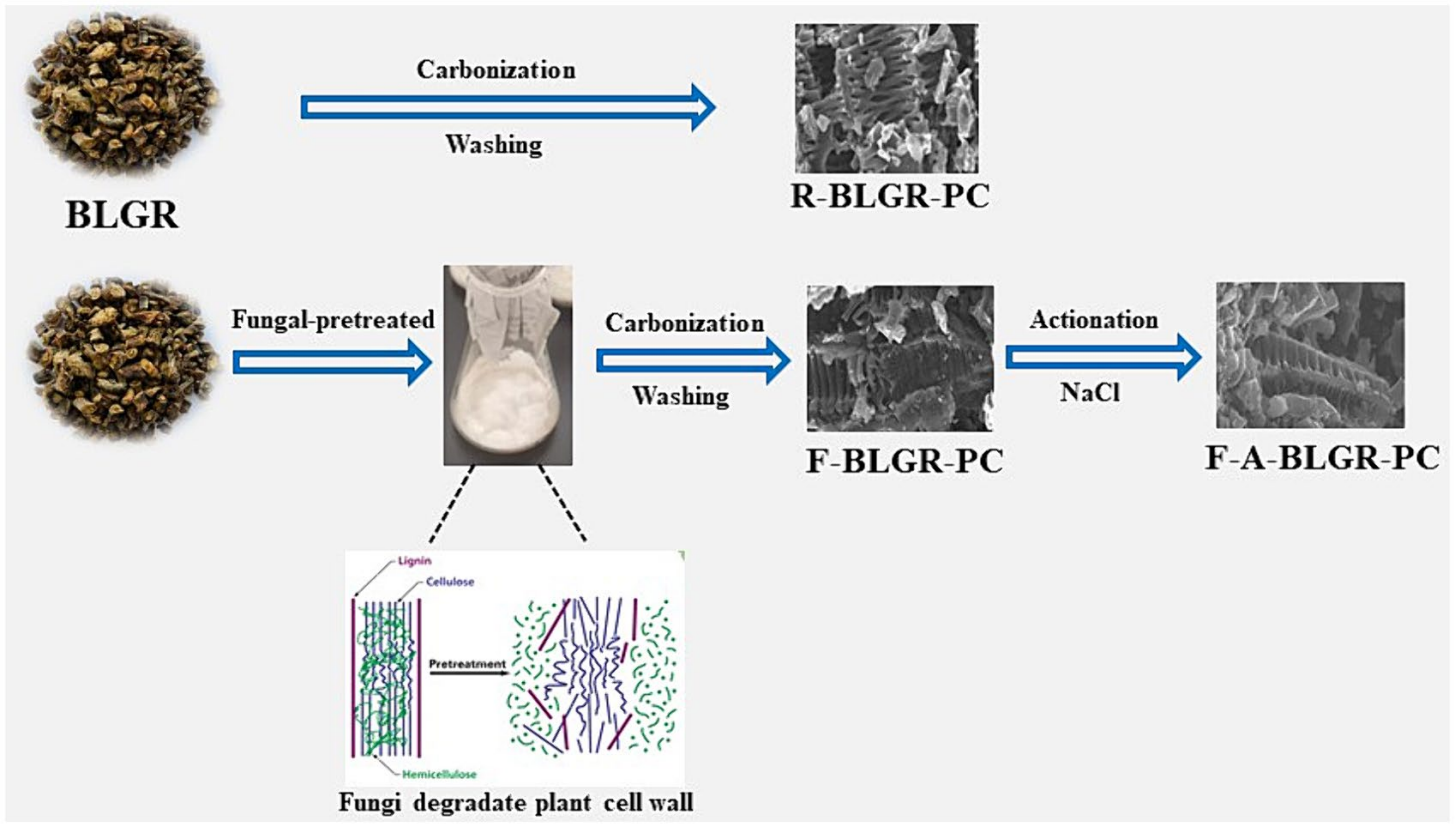
The scheme of preparation of porous carbons from BLGR.

Raw BLGR and fungal-treated one were subjected to carbonization, respectively. In a typical synthesis process, fungal-treated BLGR were placed in a tube furnace and carbonized at 700°C for 2 h with a ramping rate of 5°C min^−1^ under a continuous flow of N_2_. After cooling down to room temperature, the products were washed with 1 M HCl and deionized water several times until the pH was neutral, and then dried at 120°C for 12 h and the resultant carbon was denoted as F-BLGR-PC. As comparison, the carbon samples prepared from direct carbonization of raw BLGR was named R-BLGR-PC. The derived F-BLGR-PC was activated with NaCl as activator. In detail, the F-BLGR-PC was thoroughly mixed with NaCl (the mass ratio of biochar to NaCl was 1:2) and further pyrolyzed in a tubular furnace at 700°C for 2 h in flowing N_2_ with a heating rate of 5°C min^−1^. The schematic of the preparation of porous carbons derived from BLGR is depicted in Scheme 1.

After activation by NaCl, the residue of activator was removed by washing with dis tilled water for several times. The derived porous carbon after NaCl activation was denoted as F-A-BLGR-PC.

### 2.4 Material characterization

The morphology of the samples was characterized by scanning electron microscopy (SEM, Carl Zeiss Sigma 300). X-ray diffraction (XRD) pattern was obtained by a Shimadzu XRD-6100 X-ray diffractometer in the 2θ range of 10–80° with Cu-Kα radiation. The Raman spectra were carried out using a Renishaw Via Spectrometer. X-ray photoelectron spectroscopy (XPS) was tested on a Thermo ESCALAB 250XI spectrometer. The nitrogen isothermal adsorption and desorption curves were tested at 77 K on a Micromeritics ASAP 2020 surface area pore size analyzer. The Brunauer–Emmett–Teller (BET) formula was used to calculate the specific surface area (SSA), and Density Functional Theory (DFT) was used to calculate the pore size distribution (PSD) curve of the isothermal adsorption curve.

### 2.5 Electrochemical measurement

The electrochemical properties of porous carbon samples were tested by cyclic voltammetry (CV), galvanostatic charge–discharge (GCD) and electrochemical impedance spectroscopy (EIS) techniques on a CHI660E electrochemical workstation (Shanghai Chenhua Company, China). The cyclic stability of the electrode was evaluated by GCD curves measured on a LANHE-CT2001A test workstation. In the three-electrode system, a platinum electrode was used as an auxiliary electrode and a Hg/HgO electrode as the reference electrode. The electrode was prepared by grinding and mixing 70 wt% BLGR-PCs, 25 wt% conductive carbon black and 5 wt% polytetrafluoroethylene (PTFE) with ethanol as a dispersant. The resulting slurry was evenly spread on 1 cm^2^ nickel foam and dried overnight under vacuum at 80°C. The prepared electrode sheet was pressed under a pressure of 10 MPa. The mass loading of BLGR-PCs on the working electrode was 0.7–1.1 mg cm^−2^, and the electrolyte was 6 M KOH solution for the three-electrode configuration. The specific capacitances (Cs, F g^−1^) were determined from the GCD curves according to the following Eq. (1):


(1) $$Cs\frac{I\;\Delta \;t}{m\;\Delta \;V}$$

In the two-electrode system (symmetric device), two identical working electrodes were assembled and 1.0 M Na_2_SO_4_ solution was used as the electrolyte so that a wide working voltage can be realized. The specific capacitance (Cg, F g^−1^) of a single electrode was calculated based on Eq. 2.


(2) $$Cg\frac{4I\;\Delta \;t}{m\;\Delta \;V}$$

The values of energy density (E, Wh kg^−1^) and power density (P, W kg^−1^) of the symmetric supercapacitor were calculated by using Eqs 3, 4, respectively.


(3) $$E=\frac{\left(C\Delta V^{2}\right)}{7.2}$$

(4) $$P=\frac{3600E}{\Delta t}$$

where ΔV (V) is the cell potential change excluding voltage drop (IR drop), m (g) is the mass of active material on single electrode, I (A) is the discharge current, Δt (s) is the discharge time.

### 2.6 Adsorption measurements

The adsorption capacity of BLGR-PCs was assessed using a 50 mg L^−1^ standard solution of methyl orange (MO) in deionized water. Each adsorption test employed 100 mL of the MO solution with 0.25 g of adsorbent, equivalent to an adsorbent concentration of 0.25 g L^−1^. The mixture of adsorbent and MO solution was agitated at 200 rpm in a water bath at 25°C to facilitate adsorption. At specified intervals, 1 mL of the mixture was withdrawn, centrifuged to isolate the liquid phase, and the dye concentration in the supernatant was determined at 464 nm using a UV–Vis spectrophotometer. The amount of dye adsorbed on the adsorbent (q, mg/g)was calculated using Eq. 5:


(5) $$q=\frac{\left(C_{0}-C_{e}\right)V}{1000m}$$

Where C₀ and C_e_ (mg L^−1^) are the initial and equilibrium mass concentrations, V (mL) is the volume of the MO solution, and m (mg) is the amount of adsorbent added.

## 3 Results

### 3.1 Pore properties and morphology characterization

**Figure 1:**
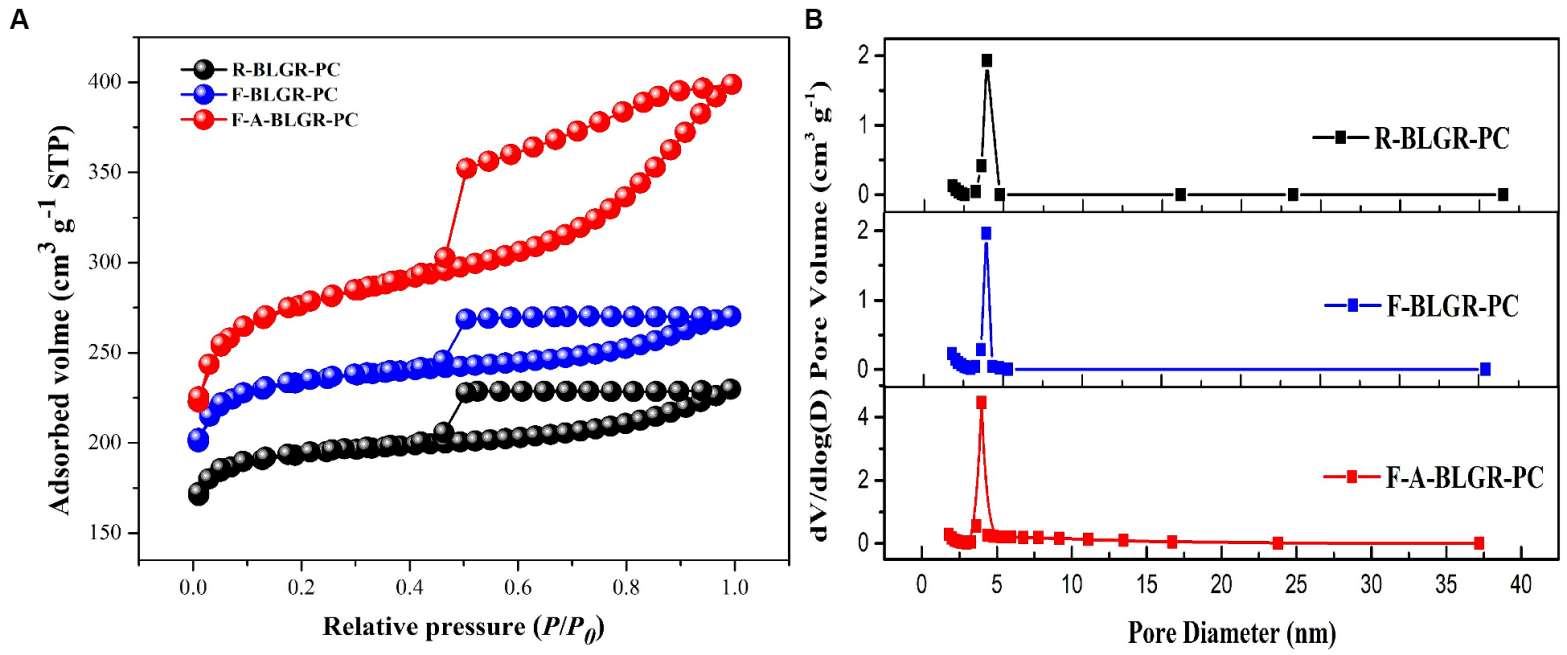
**(A)** N_2_ adsorption/desorption isotherms and **(B)** pore size distribution of the R-BLGR-PC, F-BLGR-PC and F-A-BLGR-PC samples.

Figure 1A displays the nitrogen adsorption/desorption isotherms of R-BLGR-PC, F-BLGR-PC and F-A-BLGR-PC, which are classified as a typical type IV shaped pattern according to the IUPAC classification (Thommes et al., 2015). This type of isotherm exhibits a characteristic of an obvious H4 hysteresis loop, indicating that the samples are mainly composed of mesopores. Figure 1B indicates the pore size distribution plots of the three samples, which all illustrate a mean size distribution around 4.0 nm, further confirming the existence of porous structure (Kim et al., 2019). Moreover, the sharp increase of nitrogen uptake at a relative pressure P/P_0_ lower than 0.1 implies the presence of a considerable amount of micropores. The PSD of the three samples reveals that they all possess abundant mesoporous structures and a small proportion of microporous structures.

Table 1 shows the textual parameters like the total BET surface area (S_BET_), t-plot micropore surface area (S_micro_), total pore volume (V_total_) and micropore volume (V_micro_) of the derived carbon samples. Compared to the R-BLGR-PC, the fungal pretreated materials F-BLGR-PC and F-A-BLGR-PC have a significantly higher specific surface area. Specifically, the specific surface area (SSA) of F-BLGR-PC and F-A-BLGR-PC are 741 m^2^ g^−1^ and 898 m^2^ g^−1^ respectively, while the SSA of R-BLGR-PC is low to 626 m^2^ g^−1^. The SSA of F-BLGR-PC exceeded that of R-BLGR-PC by 17.39%, demonstrating the efficacy of fungal pretreatment in increasing SSA. This suggests that white-rot fungi pretreatment is conducive to enhancing porosity by disrupting the original material structure. Moreover, the combined approach of fungal pretreatment and NaCl activation further boosts the surface area, with the SSA of F-A-BLGR-PC surpassing that of R-BLGR-PC by 43.36%.

**Table 1:** Pore parameters of the R-BLGR-PC, F-BLGR-PC and F-A-BLGR-PC samples.

Samples	S_BET_ (m^2^ g^−1^)	S_micro_ (m^2^ g^−1^)	V_total_ (cm^3^ g^−1^)	V_micro_ (cm^3^ g^−1^)	V_micro_/ V_total_	S_micro_/ S_BET_
R-BLGR-PC	626	539	0.64	0.30	46.88%	86.05%
F-BLGR-PC	741	623	0.77	0.36	46.75%	84.08%
F-A-BLGR-PC	898	673	0.97	0.38	39.18%	74.98%

**Figure 2:**
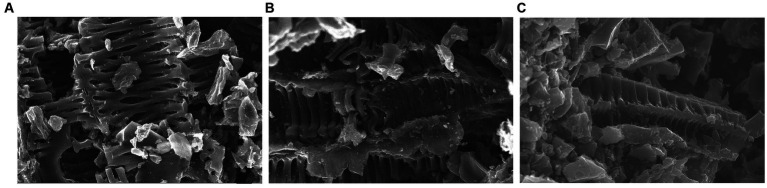
SEM images of **(A)** R-BLGR-PC; **(B)** F-BLGR-PC; **(C)** F-A-BLGR-PC.

Scanning electron microscopy (SEM) was used to further investigate the morphology evolution of the resultant products. The results in Figure 2 show that the non-inoculated sample R-BLGR-PC has a few pores and crevices on its surface, but with intervals between the pores (Figure 2A). The F-BLGR-PC sample owns a rib-shaped structures on its surface due to enlarged pores and crevices (Figure 2B). Whereas, the F-A-BLGR-PC shows a much looser surface structure with abundant pores (Figure 2C). This phenomenon can be explained by the fact that white-rot fungi can degrade plant cell wall components and create an internal loose structure, allowing the NaCl template to access the internal surface of the carbons and undergo activation more effectively (Haneef et al., 2017; Wang et al., 2019). The above results suggest that carbon materials fabricated by pretreating the starting material with white-rot fungi have a much looser structure with visible pores than that of pretreatment-free ones. The resultant BLGR material owns the loosest structure, indicating that the white-rot fungi pretreatment plays a key role in the synthesis of the AC.

### 3.2 Crystallinity analysis

**Figure 3:**
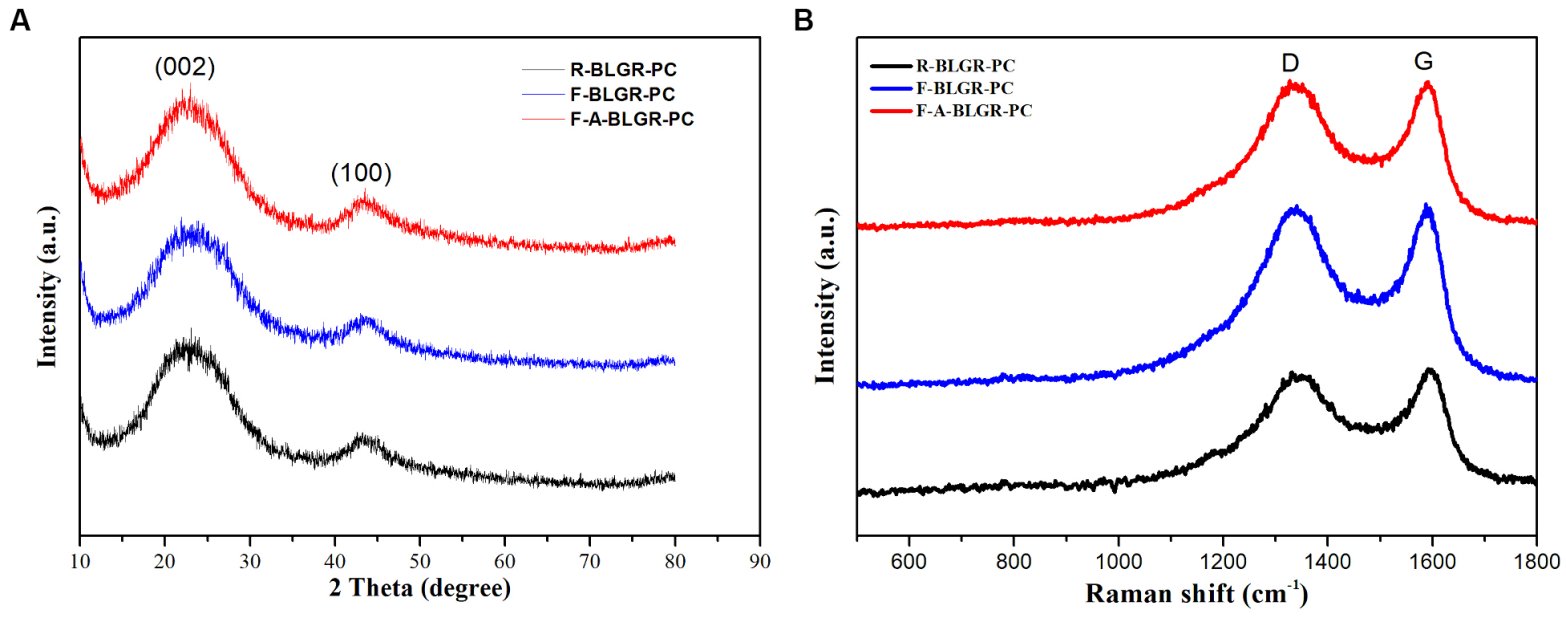
**(A)** XRD patterns; **(B)** Raman spectra.

As illustrated in Figure 3A, the XRD patterns of the three samples are similar, featuring two wide peaks at around 24° and 44°, which corresponds to the (002) and (101) crystal planes of the graphite structure. This implies that the original BLGR biomass has been graphitized and its main component is amorphous carbon (Gaddam et al., 2016). Figure 3B presents the Raman spectrum with two prominent peaks, namely, the D-band at 1340 cm^−1^ and the G-band at 1590 cm^−1^, symbolizing irregular vibration and sp^2^ hybridized carbon with a graphite structure, respectively (Dutta et al., 2014). The graphitization degree of the material can be assessed through the I_G_/I_D_ ratio of the G-band to the D-band. The higher I_G_/I_D_ ratio means the better the graphitization degree. The calculated I_G_/I_D_ values of R-BLGR-PC, F-BLGR-PC and F-A-BLGR-PC are 1.01, 0.99 and 0.98, respectively. It is evident that the graphitization degree of the F-A-BLGR-PC is lower than that of R-BLGR-PC and F-BLGR-PC, which can ascribed to the generation of more mesopores in F-A-BLGR-PC. The presence of mesopores prevents the parallel arrangement and binding of graphitic microcrystals, thus impeding the further growth of graphite microcrystals.

**Figure 4:**
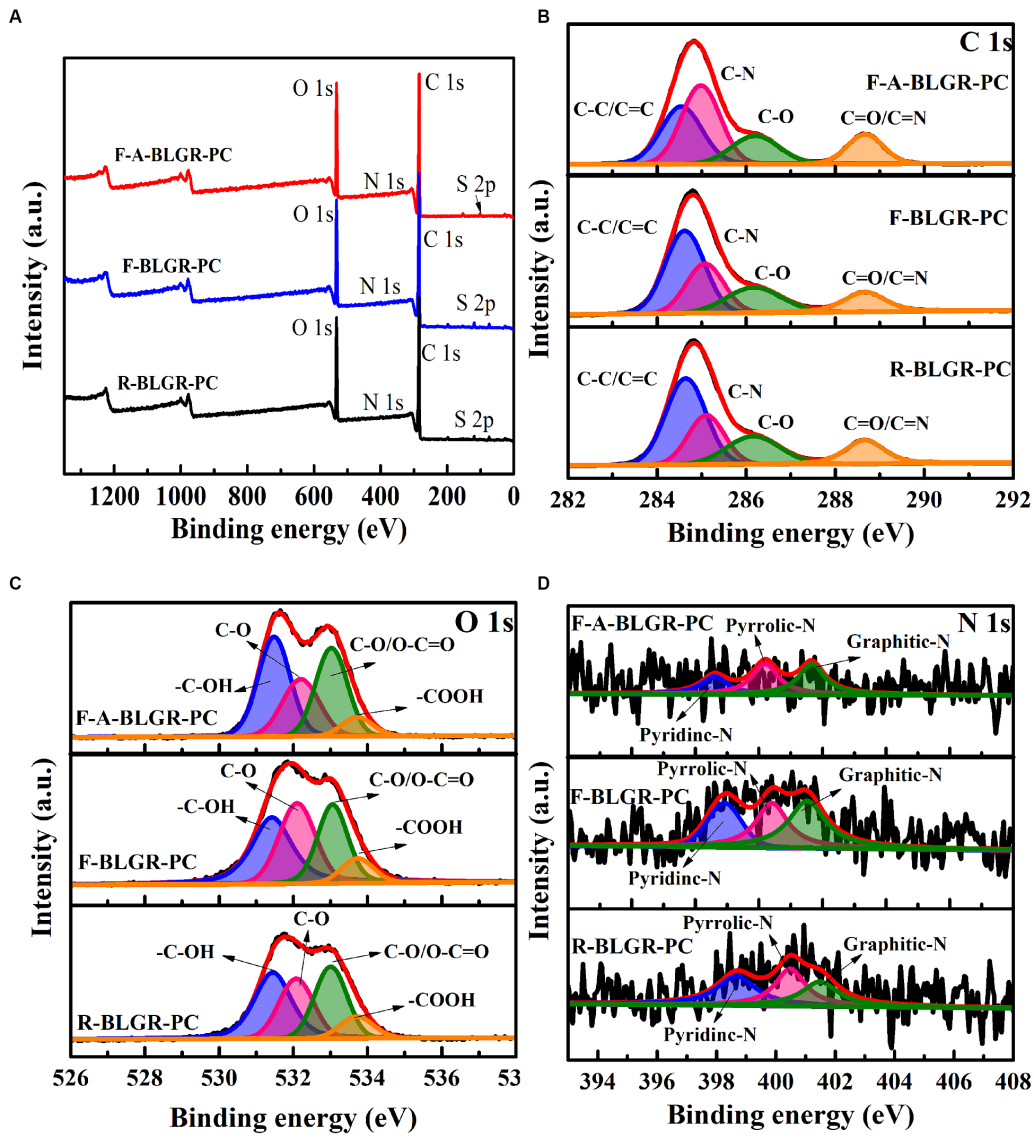
**(A)** High-resolution XPS survey spectra, **(B)** C1s, **(C)** O1s and **(D)** N1s spectra of the R-BLGR-PC, F-BLGR-PC and F-A-BLGR-PC samples.

X-ray photoelectron spectroscopy (XPS) was employed to investigate the chemical composition and surface chemistry of BLGR-PCs. The full scan XPS spectra in Figure 4A reveals the presence of C, N and O species (Jiang W. et al., 2019). The detailed contents of the three elements are depicted in Table 2 in supporting information. High resolution C1s spectra shows four peaks at 284.65, 285.11, 286.16 and 288.66 eV (Figure 4B), which corresponds to C–C/C=C, C–N, C–O and C=O/C=N speices, respectively (Xu et al., 2016; Zhou et al., 2019). This C-N bond indicates the incorporation of nitrogen elements into the carbon skeleton (Shao et al., 2023). After fungal pretreatment and activation, the content of C-N bond increased from 23.11 to 38.67% (Table 2). It was proved that the incorporation of nitrogen into porous carbon materials could enhance the wettability and conductivity, leading to an increased pseudocapacitance (Zhao et al., 2017). N1s spectra showed four peaks at 398.66, 400.47, and 401.48 eV, which are attributed to pyridinic-N, pyrrolic-N, and graphitic-N, respectively (Figure 4D). The white rot fungus pretreatment and activation have been observed to increase the content of graphic-N from 28.51 to 38.65% (Table 2), which has been beneficial in terms of decreasing the electron transfer resistance and enhancing the electrical conductivity (Liu et al., 2019). Figure 4C shows the O 1 s region spectrum, which includes four peaks situated at 531.45, 532.08, 533.01 and 533.71 eV corresponding to the C–OH, C–O, O–C = O/C = O, and COOH functional groups, respectively, (Zhou et al., 2019). The oxygen-containing functional groups in porous carbon can create numerous defects, resulting in the formation of numerous active sites (Jiang S. -F. et al., 2019).

**Table 2:** The content of elemental compositions of R-BLGR-PC, F-BLGR-PC and F-A-BLGR-PC samples.

Sample	C^a^ (atom%)				N^a^ (atom%)			O^a^ (atom%)			
	C-C/C=C^a^	C-N^a^	C-O^a^	C=O/C=N^a^	Pyridinic-N^a^	Pyrrolic-N^a^	Graphitic-N^a^	C-OH^a^	C-O^a^	C=O^a^	-COOH^a^
R-BLGR-PC	77.62				0.88			21.50			
	44.59	23.11	19.29	13.01	35.28	36.22	28.51	34.95	26.29	29.42	9.34
F-BLGR-PC	77.35				1.02			21.63			
	43.90	25.10	18.89	12.12	26.82	33.08	40.1	33.29	31.86	26.59	8.25
F-A-BLGR-PC	77.95				0.64			21.41			
	30.81	38.67	17.04	13.47	22.58	38.76	38.65	37.46	24.03	31.75	6.75

a The content of various elements was determined by the XPS measurement.

### 3.3 Electrochemical performance

**Figure 5:**
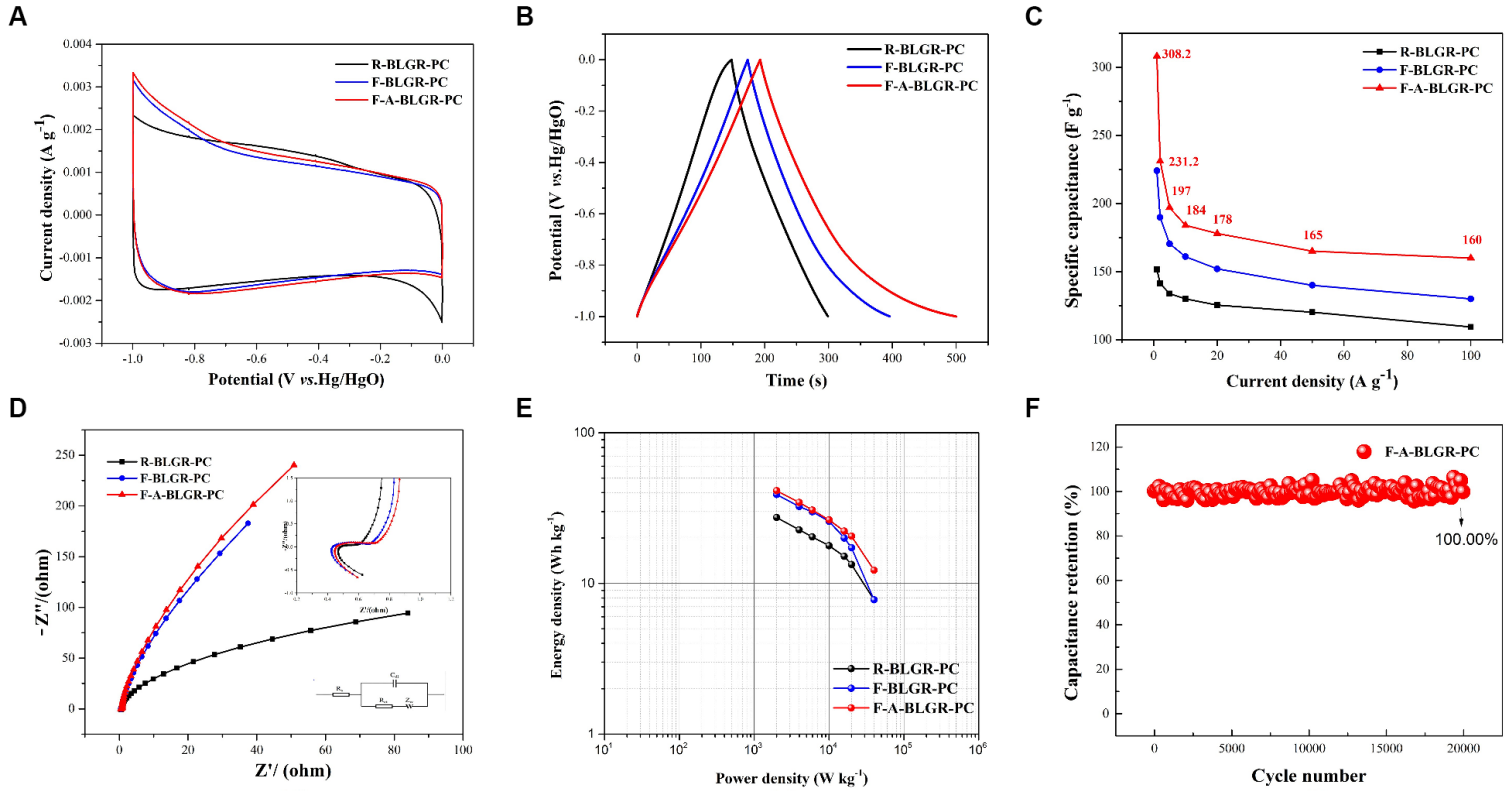
Electrochemical performance of the BLGR-PCs electrodes tested in 6 M KOH electrolyte: **(A)** CV curves at a scan rate of 10 mV s^−1^; **(B)** GCD curves at a current density of 1.0 A g^−1^; **(C)** Specific capacitance versus current density; **(D)** Nyquist plots of the electrodes and the inset is the magnified image of high-frequency region; **(E)** Ragone plots (energy density versus power density) for BLGR-PCs; **(F)** Cycling stability at a current density of 5 A g^−1^ for the F-A-BLGR-PC.

Utilizing a three-electrode system with 6 M KOH as electrolyte, the energy storage performances of R-BLGR-PC, F-BLGR-PC and F-A-BLGR-PC were assessed. The CV curves for the three specimens all display a quasi-rectangular form in the potential range of −1.0 to 0 V, indicating that the capacitances of the three electrodes was contributed by electric double layered capacitance (EDLC) and Faradaic capacitance (Figure 5A). At current density of 1.0 A/g, the GCD curves of all samples maintain a symmetrical shape, indicating a low resistance, good reversibility, and high coulombic efficiencies (Figure 5B). Notably, the GCD curve of F-BLGR-PC and F-A-BLGR-PC was slightly distorted, indicating the presence of pseudocapacitance, which is attributed to the reversible Faradaic reaction of the introduced nitrogen- and oxygen-containing functional groups in F-BLGR-PC and F-A-BLGR-PC with electrolyte (Ji et al., 2016; Liu et al., 2017). Calculations based on the discharge curves (0.5 A/g) yields capacitances of 152, 224, and 308F/g for R-BLGR-PC, F-BLGR-PC and F-A-BLGR-PC, respectively (Figure 5C).

The electrochemical impedance spectroscopy (EIS) technique was employed to study the diffusion and transport kinetics of electrolyte ions. The Nyquist plots of the electrodes, as illustrated in Figure 5E, display a semicircular pattern with charge transfer resistance (Rct) and volume resistance (Rs). In high-frequency regions, the Rs values of R-BLGR-PC, F-BLGR-PC, and F-A-BLGR-PC electrodes were 0.625, 0.563, and 0.593 Ω, respectively. As Rs represents the conductivity of the electrode material, it can be inferred that the pretreatment and activation of white rot fungus could increase the conductivity of F-BLGR-PC and F-A-BLGR-PC. As demonstrated by Figure 5E, F-A-BLGR-PC has a considerably higher energy and power density than the other samples, making it highly advantageous in practical applications. Furthermore, the galvanostatic charge–discharge (GCD) measurement was used to evaluate the cycle stability of F-A-BLGR-PC at 5.0 A/g, which indicates an impressive capacitance retention of near 100% after 20,000 cycles (Figure 5F).

### 3.4 Methyl orange adsorption properties

**Figure 6:**
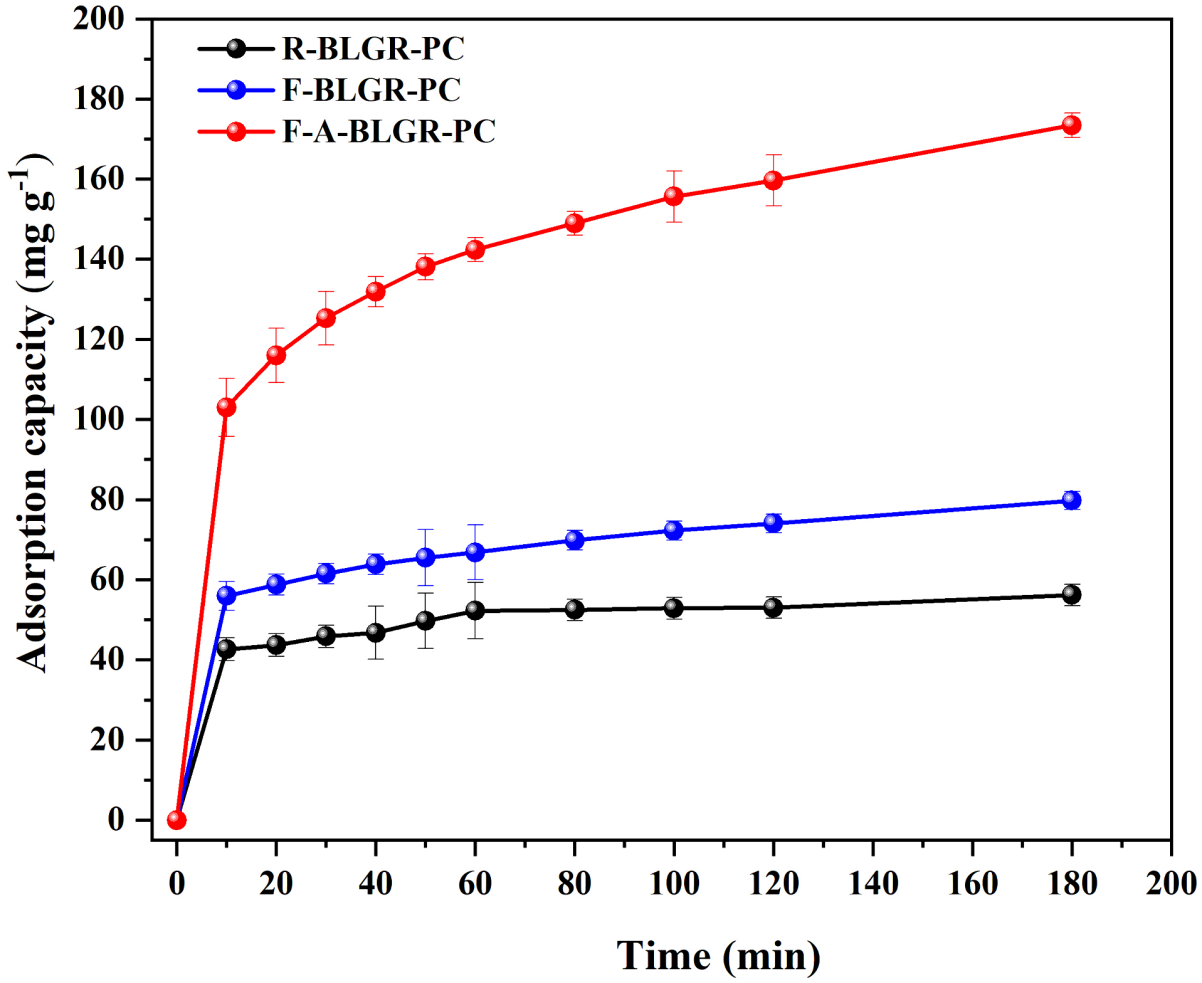
Adsorption performance of R-BLGR-PC, F-BLGR-PC and F-A-BLGR-PC: 50 mg/L of MO, 12.5 mg (0.25 g/L) of samples, 25°C.

Figure 6 illustrates the adsorption of Methyl orange (MO) on different carbon samples measured at room temperature. It is evident that sample F-A-BLGR-PC has the most effective adsorption of methyl orange with an equilibrium adsorption capacity of 173.42 mg/g and a maximum removal rate of 86.55%. Obviously, the superior adsorption capacity of F-A-BLGR-PC suggests that fungi pretreatment is beneficial for improving the adsorption capacity of MO. This is in line with the data in Table 1, as higher BET surface area and pore volume were obtained after fungi pretreatment.

## 4 Discussion

The utilization of biomass-derived carbon-based materials is gaining increasing attention in recent years. The large amount of traditional Chinese medicine residue is a major environmental concern. In this study, we explored the potential of using traditional Chinese medicine residue as a carbon source for the production of porous carbon with versatile functionalities. We employed a biological pre-pore-forming method, inspired by the degradation of biomass by white rot fungi in nature, to prepare porous carbon with a high specific surface area. By leveraging the special degradation capabilities of *P. vitreus*, we were able to obtain high-performance F-A-BLGR-PC through a simple carbonization and NaCl activation process of Banlangen residues.

The comparison between porous carbon prepared directly from raw biomass and the ones prepared from biomass pretreated by *P. vitreus* reveals that the latter exhibits higher porosity and larger specific surface area. This is attributed to the white rot fungi treatment, which introduces enzymes that degrade the lignin and hemicellulose components of the biomass, leading to a more open and porous structure. This facilitates the access of activation agents during carbonization and activation, resulting in higher porosity (Ding et al., 2012; Wang et al., 2016; Wu et al., 2021). SEM results suggest that F-BLGR-PC based products are much looser in structure than R-BLGR-PC. Raman spectra further confirms that F-A-BLGR-PC has the lowest graphitization degree due to its high porosity. It is clear that both fungal pretreatment and NaCl activation are essential for the development of porosity in carbon.

Research has demonstrated that the white rot fungi possess superior infiltrability and biodegradability of their hyphae towards lignocellulose, making them a potential option for the construction of artificial hierarchical architectures (Jiang S. -F. et al., 2019; Shao et al., 2020, 2023). As the main biomass degrading organisms, white rot fungi are responsible for the decomposition of lignin in nature (Floudas et al., 2012; Shao et al., 2020, 2022a). This process involves the formation of a 3D network of hyphae. The mycelium of these fungi spreads quickly in three-dimensional space along the cell walls of plants, and has a strong mechanical interpenetrating capacity. After being degraded by white rot fungi, traditional Chinese medicine residues become softer and more pliable, creating a favorable microbial “pre pore” for the preparation of ultra-high specific surface area porous carbon materials. Fungi have the potential to generate layered porous structures. By taking advantage of their biodegradation abilities and filamentous permeability, loosely structured lignocellulosic precursors can be formed, which is appropriate for effective carbonization and chemical activation (Wang et al., 2019; Zhang et al., 2019). The looser structure after fungal pretreatment allows for a more thorough activation, leading to a higher specific surface area compared to untreated samples. This is attributed to the ability of the fungi to create an interconnected pore network, enabling more efficient carbonization and activation. The fungal pretreatment introduces mesopores and microspores into the structure. This balanced pore distribution contributes to the high specific surface area, with the mesopores providing fast ion transport pathways. As a result, traditional Chinese medicine residues can be softened and loosened after degradation, which is beneficial to the preparation of porous carbons with high specific surface areas.

The electrochemical performance results of F-A-BLGR-PC indicate that it has the characteristics of EDLC and Faradaic behavior. Moreover, the F-A-BLGR-PC demonstrats a highest adsorption capacity for methyl orange in an aqueous solution ascribed to its large surface area and pore volume. This is supported by the data listed in Table 1 which shows that the higher BET surface area and pore volume corresponds to a greater adsorption capacity (Chen et al., 2023; Gao et al., 2024). It is clear that the porous structure created by the fungal pretreatment significantly enhances the adsorption performance of the prepared carbon resulted from the white rot fungi infiltrating and biodegrading the internal structure of the biomass substrate, thereby increasing the efficacy of the carbonization and chemical activation steps (Ding et al., 2012; Wang et al., 2019; Zhang et al., 2019). The introduction of nitrogen and oxygen-containing functional groups by the fungi pretreatment enhances the pseudocapacitance contribution to the overall capacitance, leading to higher specific capacitance values compared to untreated samples. The high surface area, rich oxygen functionalities, and pseudocapacitance from the introduced nitrogen/oxygen groups contribute to the high specific capacitance and excellent cycle stability of the fungal pretreated carbon. The improved ion transport and reduced charge transfer resistance further enhance the electrochemical performance. The balanced pore distribution and interconnected pore network provide an optimal environment for electrolyte ion diffusion and transport, facilitating rapid ion access and adsorption/desorption processes.

The produced porous carbon has a high specific surface area and may be suitable for a variety of applications. This research not only provides a way to make use of traditional Chinese medicine residues by transforming them into valuable carbons, but also serves as a source of inspiration for optimizing the performance of porous carbons. Further optimization of the conditions of the porous carbon produced from fungi-pretreated biomass could be explored to enhance its performance. Additionally, further research is needed to investigate the compatibility between fungi type and biomass nature in the fabrication of porous carbon.

## 5 Conclusion

In summary, a white rot fungi pretreatment method was explored and applied to the synthesis of porous carbon from BLG medicinal residue. The white rot fungi pretreatment method effectively softens and loosens the structure of the Radix isatidis residue, facilitating the creation of a highly porous porous carbon with enhanced properties. The resultant F-A-BLGR-PC exhibits a SSA of 898 m^2^ g^−1^, which is 43.36% greater than that of the unprocessed R-BLGR-PC sample. As electrode for supercapacitor, the maximum specific capacitance of 308F g^−1^ at 1 A g^−1^ was achieved for F-A-BLGR-PC. Moreover, the adsorption of methyl orange test show a maximum removal rate of 86.55% of the R-BLGR-PC was achieved. The results indicates that the performances including supercapacitor and sorption of the resultant carbon materials treated with microorganisms was significantly enhanced as compared to that of the untreated porous carbon materials. Importantly, the present research offers an alternative route to prepare porous carbon from biomass waste in a biological way and the resultant product may also find applications in many other fields like catalysis, separation, and so on.

## Data availability statement

The original contributions presented in the study are included in the article/supplementary material, further inquiries can be directed to the corresponding author.

## Author contributions

WK: Conceptualization, Formal analysis, Funding acquisition, Methodology, Software, Supervision, Validation, Writing – original draft, Writing – review & editing. XZ: Data curation, Formal analysis, Investigation, Methodology, Software, Writing – original draft. XF: Data curation, Methodology, Writing – original draft. CZ: Data curation, Investigation, Methodology, Writing – original draft. LF: Data curation, Methodology, Writing – original draft. WZ: Conceptualization, Formal analysis, Funding acquisition, Investigation, Project administration, Resources, Validation, Writing – original draft, Writing – review & editing.
